# Knowledge mining of unstructured information: application to cyber domain

**DOI:** 10.1038/s41598-023-28796-6

**Published:** 2023-01-31

**Authors:** Tuomas Takko, Kunal Bhattacharya, Martti Lehto, Pertti Jalasvirta, Aapo Cederberg, Kimmo Kaski

**Affiliations:** 1 Department of Computer Science, Aalto University School of Science, 00076 Espoo, Finland; 2 Department of Industrial Engineering and Management, Aalto University School of Science, 00076 Espoo, Finland; 3 Faculty of Information Technology, University of Jyväskylä, PO Box 35, 40014 Jyväskylä, Finland; 4 Cyberwatch Finland, Meritullinkatu 33, 00170 Helsinki, Finland; 5 The Alan Turing Institute, 96 Euston Rd, King’s Cross, London, NW1 2DB UK

**Keywords:** Computational science, Computer science, Information technology

## Abstract

Information on cyber-related crimes, incidents, and conflicts is abundantly available in numerous open online sources. However, processing large volumes and streams of data is a challenging task for the analysts and experts, and entails the need for newer methods and techniques. In this article we present and implement a novel knowledge graph and knowledge mining framework for extracting the relevant information from free-form text about incidents in the cyber domain. The computational framework includes a machine learning-based pipeline for generating graphs of organizations, countries, industries, products and attackers with a non-technical cyber-ontology. The extracted knowledge graph is utilized to estimate the incidence of cyberattacks within a given graph configuration. We use publicly available collections of real cyber-incident reports to test the efficacy of our methods. The knowledge extraction is found to be sufficiently accurate, and the graph-based threat estimation demonstrates a level of correlation with the actual records of attacks. In practical use, an analyst utilizing the presented framework can infer additional information from the current cyber-landscape in terms of the risk to various entities and its propagation between industries and countries.

## Introduction

The cyberspace is increasingly facing challenges in the form of persistent and devious threats from state and non-state actors alike. Given the growth of smart devices, data storage options, and supply-chain dependencies, establishing the security and resiliency in the cyberdomain has become an imperative for companies and organizations across sectors^1^. A key challenge here is to assimilate the large-scale data in free-forms, such as reports on incidents and vulnerabilities that are openly available from numerous sources including vulnerability databases and international agencies^2^. An ad-hoc structuring of information by interlinking reports on events, i.e. a knowledge graph framework^3–6^, appears to be a viable solution. The concept of knowledge graphs has been adopted for structuring and processing of technical information on known vulnerabilities, malicious IP addresses and different relevant threats in the cyberdomain, as well as for associating other related entities such as software companies.

In this article we present a computational framework for analyzing the cyber-landscape by utilizing publicly available textual reports of various incidents. The objective is to get a broad yet condensed view of the relevant interconnected entities and the prevailing threats. In addition to a visual tool, we explore the usability of the resulting knowledge graph in terms of estimating the risk of cyberattacks. Such frameworks have been proposed in the past, but only a few studies provide methods for practical and automated construction of visual and graph-based solutions. The framework is based on high level descriptions of cyber-incidents, like attacks and breaches, which can easily be obtained from open online sources.

This work shares a broader objective similar to the work of Böhm et al.^7^, in which the authors described and justified a human-readable and visual approach for analyzing complex cyberattack reports. The success of security experts depends on the readability of available intelligence, information in structured formats and ontologies, which often require additional tools and frameworks for actionable usage. In general, ontologies such as STIX^8^, UCO^9^, and STUCCO^10^ consist of various technical or higher level entities and their possible interrelations that facilitate the interlinking of entities and events. While these ontologies have excelled in focusing on the microscopics and different technical sophistication, there still remains ample scope to portray the cyber-landscape in a clear and readable manner for the ease of analysis and subsequent decision making at a strategic level.

In the vein of earlier research^6,7,11^ we extend the knowledge graph constructed from unstructured data by joining information from separate other sources of data. We use crawling and querying for additional records and information about the entities from sources such as DBpedia^12^. The additional information is aimed to sufficiently populate the ontology and to enhance interconnectedness in the knowledge graph , which subsequently allows us to calculate the risk. Furthermore we demonstrate that the graph can be used to determine a risk level for the entities in the graph by using historical data on cyberattacks. This risk level could be used to estimate the likelihood of future cyberattacks given the past incidents on connected entities.

This article is structured in the following manner. In “Related work” section, we establish the position of the current framework in relation to existing works and studies in the field of open source and knowledge graph based systems, by focusing on the studies that have overlapping methods or data sources. Next, in Methods and Materials (“Methods and materials” section) we describe the processing pipeline for producing a strategic level knowledge graph from unspecified textual sources, such as news reports on cyberattacks. In “Results and analysis on an experimental dataset” section, we analyze the knowledge graph and use it to measure a type of risk. Finally, in “Discussion” section we discuss the relevance of our findings in terms of their usefulness to cyber-analysts and enumerate the limitations. In “Conclusions” section we summarize our findings and discuss the possible future improvements.

## Related work

The concept of knowledge graph, where complex information is represented as nodes with edges as semantic relations between them^13,14^, has become increasingly popular in numerous fields of research and in the implementation of information-driven applications. Improved methods for extracting meaningful information and entities from unstructured text^15^, as well as the increasing coverage of linked data from various endpoints (such as DBpedia^12^) has made it possible to query for relevant information and to connect information from text to existing records of various entities, events and items. The applications of knowledge graph ranges from systems in healthcare^16,17^ to search systems and scientific document indexing^8,16–18^.

In cybersecurity and cyber intelligence, the use of knowledge graphs and linked data has been prevalent due to the mostly structured nature of the recorded data related to intrusion detection systems, software vulnerabilities and malicious actors^3,19^. For instance, online databases like NVD^20^, CVE^21^ and CWE^22^ provide regular updates on software and system vulnerabilities in a structured format. Cyberdefense benefits from synergy and cooperation, but sharing and interpreting various threat intelligence reports and databases requires standardized formats and protocols for the analysts to have a common language^23^. Thus, there has been extensive research done for constructing taxonomies and ontologies to standardize the formats of linked data on threat intelligence such as software and system vulnerabilities, malware, and attacks in general^8,9,24^. Using these types of ontologies to provide formalism and structure, various framework-type approaches to situational cyber awareness have been developed, for instance for different vulnerabilities, assets and network topologies during cyberattacks^6,7,25–27^. Other approaches for extracting relevant information on cyberattacks and vulnerabilities from different unstructured text sources, such as social media, have been used as early warning signals for cyber-risks^28–33^.

The present study is related to earlier works^6,11,34^ that describe and implement methods for a pipeline with the objective to turn unstructured data into knowledge graphs, based on specific ontologies. For instance, Joshi et al.^11^ described a framework that processes unstructured web text from security bulletins and blogs alongside the vulnerability data from the NVD, CVE and CWE datasets, recognizes the entities and concepts, and finally connects them by using information from DBpedia Spotlight. In another work, Li et al.^4^ proposed a method for building a knowledge base with similar rules and structure. But instead of considering the software and hardware vulnerabilities, the ontology used device properties, attack properties and attack features. The datapoints were gathered from the network level information using a neural network classifier. Third study relevant to the scope of the current study, by Kejriwal and Szekely^34^, describes an information extraction method for unstructured text, scraped from illicit web domains. The authors proposed methods for annotating and extracting information such as entities and locations using unsupervised methods based on an initially annotated corpora.

The framework presented in this paper shares principle level similarities to the studies described above, in terms of the structure of the data processing pipeline and methods. This work shares similarities to the general objective of the work by Li et al.^6^ in portraying cyberattacks in a knowledge graph format. The latter framework processes data from the network and information systems of an entity, whereas the strategic domain approach of this study is restricted to open source information from security bulletins and news sources, thus limiting the number of features and amount of information available. The information extraction part of this study has principles similar to the work of Kejriwal and Szekely^34^, with the objective of processing information from unknown domains and extracting the relevant entities and their relationships. We extract the entities using a named entity recognizer (NER) from spaCy^35^ and compare the extracted relevant entities to the knowledge base of DBpedia using DBpedia Spotlight, similar to Joshi et al.^11^. While sharing some similarities with previously described work, the framework in this study is dependent only on the most surface level descriptions of the events, which can be acquired from easily available sources online.

## Methods and materials

**Figure 1:**
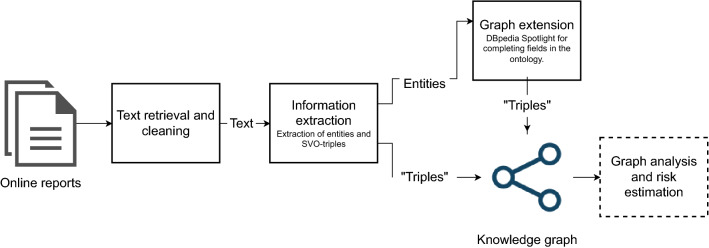
Process pipeline of the proposed knowledge mining framework. The framework and the modules are depicted as boxes with the correct order. The pipeline retrieves, cleans and extracts information from unstructured text and computes graph-level features for the analyst to investigate in the final knowledge graph.

Framework for processing unstructured information consists of three distinct modules, namely an information retrieval module, an information extraction module, and finally, a module for risk measurement and graph analysis. The framework implementation does not reuse the source material or otherwise infringe the copyright of the authors of the original text. The process with the corresponding modules is shown in Fig. 1. The first module aims to gather and process relevant unstructured information from unspecified online sources. It begins by collecting a list of urls of news reports of cyberattacks that are of interest to the analyst. The module proceeds by requesting the page from the given url, if the source allows software agents, and cleaning the obtained text by removing irrelevant content such as html-tags, other urls or embedded content. This cleaned text is then processed by removing stop words and extracting relevant entities and their relationships in the information extraction module. The relationships between the target and the attacking entities are extracted as a triple in the form of “target–attackedBy–attacker”. The extracted entities are compared to the results of DBpedia Spotlight^36^, which finds related records in DBpedia^12^ as linked data, which we then use to complete the fields in the ontology. DBpedia Spotlight annotates the entities found in the text and performs disambiguation using the context of the phrases. In an ideal situation, these entities are correctly resolved and found in DBpedia, but in a situation where this additional information is not found, we omit the information while keeping the entity as it was recognized by the spaCy NER^35^ and adding the triple of attacker–victim relationship. In a complete system one could also crawl other sources for additional information, such as software vulnerabilities. Lastly, we use the generated knowledge graph for estimating a measure for risk. The risk level in this study is based on the frequency of attacks in the connected entities of the knowledge graph.

These modules were implemented in Python 3.7 using libraries for web scraping, spaCy^35^ for information extraction and NetworkX^37^ for graph analysis. For the purpose of demonstrating our approach in this study, we opted to use the openly available datasets of cyberattacks from Hackmageddon^38^ containing reported attacks from year 2017 to 2020. The human-annotated dataset contains descriptions of targets, attackers, attack types, dates, countries and links to the original reports, which we use to obtain the full text report. The remaining fields are used in the evaluation of information extraction methods of this framework as well as to substitute for missing relationships from DBpedia. In a real use-case an analyst would use their own news sources or knowledge bases and use the framework via a user interface, or other applicable method. For the sake of clarity we restrict the number of cases analyzed in the knowledge graph to the contents of the Hackmageddon dataset. Some organizations might experience attacks or preparations of an attack on a daily basis, but those are not reported in the news whether due to their commonality, minor damage or because the organization is not releasing the information. The attacks recorded in this set of data are the ones where the attack itself is already operational and deemed news-worthy.

### Information extraction

The primary aim of this module is to identify the victim and the perpetrator of an attack from a given piece of text. The method used here is an unsupervised one^39^, and generally belongs to the category of relation extraction methods, which are used for constructing knowledge graphs in the cyber domain^40^. The approach comprises of extraction of subject–verb–object (SVO) triples, scoring for named entities, and ranking of entities. The SVO triples are extracted using a mixture of rule based methods^41^ and parsing of the dependency tree^42^. Below we will broadly describe different components in this approach. The finer details of the method and related concepts will be reported elsewhere.

1. First, the subjects and objects are tagged using spaCy noun phrases (noun-chunks).
2. The verb phrases are identified as the most general pattern: particle + adposition + verb/auxillary + particle + adposition + adjective/adverb + adposition. Similarly, lone adpositions, adverbs and adjectives are tagged. To take into account complex predicates, light verb constructions that include nouns are also recognized, for example, the phrase ‘gained access to’^43^. Additionally, Hearst patterns^44^ are identified from a pre-compiled list and using the patterns nouns are linked by using the dependency tree structure parsed by spaCy.
3. A coarser dependency tree is constructed using the noun and the verb phrases and the original dependency structure. Note, that the dependency parsing may vary depending on the language model used by spaCy. Using this coarser tree and considering the subject–verb–object order we generate the triples. The tree is parsed such that the conjugated verbs are crawled and associated with all the subjects and objects.
4. A co-reference resolution for the set of noun phrases is performed using the package NeuralCoref^45^.The resulting output from the resolution are clusters of noun phrases, where each cluster implies a single co-referenced mention.
5. Named entities are tagged using the spaCy named entity recognizer.
6. A map between the named entities in the text and the clusters from co-reference resolution is created. Using the map the subjects and the objects in the triples are replaced with the named entities.
7. For each named entity a ‘target score’ is calculated in the following fashion. A list of attack tokens is considered. The list is populated with a set of seed tokens, such that ‘hacked’, ‘breached’, etc., and further are extended by including the inflections. Given an SVO triple we check for the presence of an attack token inside the verb phrase. If a token is found and the triple has an active voice then the entity corresponding to the object gets its target score incremented by $+1$. If the voice is passive, the target score corresponding to the entity in the subject is incremented. The process is repeated for all the triples and the final target scores are obtained.
8. The number of occurrences of each entity and the order in which they appear in the text are also calculated. For each entity, a compound score is calculated by adding the min–max normalized values of the target score, the frequency of appearance, and the order (reversed). The entities are ranked in descending order of the compound score. The topmost identity is identified as the primary target mentioned in the text.

**Figure 2:**
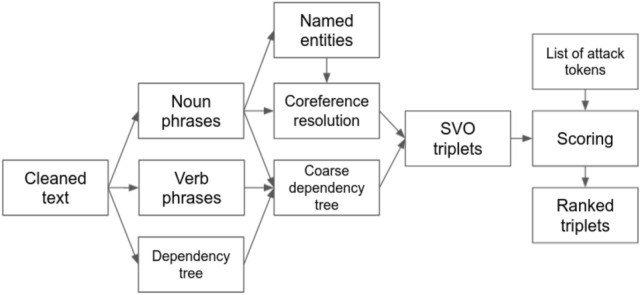
The components of the process of information extraction are shown. A major part of the process is implemented using the Python spaCy library.

The above method is illustrated in Fig. 2. We find that this method yields an accuracy of 60$\%$ for the top-most ranked entity to be the true target. However, the accuracies for the true target to be in the top-2 and top-3 ranked entities are 75$\%$ and 83$\%$, respectively. If solely the frequency or the order of appearance is taken into account the accuracy for the true target to be in the top-most and top-3 entities are around 50$\%$ and 70$\%$, respectively. Note that in general a news piece has 10–20 entities, and therefore, a baseline accuracy would be much lower in comparison. For determining the attacker, we follow a scoring method similar to item 7 in the list above by choosing the subject (object) in a triple when the voice is active (passive). However, for most of the instances in the dataset the identity of the attacker is not known. Therefore, in this case the accuracy can not be ascertained. In our future work we will provide methods whereby models can be trained on linguistic features, and quantities like frequency and order.

### Domain ontology structure

**Figure 3:**
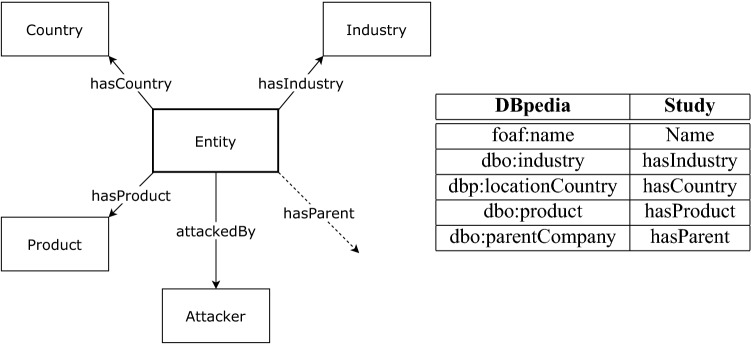
Structure of the novel strategic level cyber ontology. Subjects and objects are entities (boxes) that are connected via their relative predicates (arrows). The labels tell the class names in the ontology. The hasParent relationship is from an entity to another entity. The table lists the similarities between the predicates of the extraneous data from DBpedia and the corresponding predicates ontology in this study.

The framework of this study utilizes a novel domain ontology for defining the elements and the relationships appearing in the knowledge graph. The ontology is depicted in Fig. 3. It captures knowledge on the entities and actors at a strategic level, i.e. at a level that describes real world structures and helps in constructing a broader picture of the whole field at once. The extracted information for each report on a cyberattack depicts the main attributes of an organization and ideally forms a connected network, in which visualizing trends and campaigns along with individual attack incidents is possible. The entities, such as companies and organizations, are described by their countries and industries as well as by their products and possible child–parent relationships to other entities. Different countries, products and industries appear in the knowledge graph as nodes alongside the organizations and attacking entities. We categorize industry and country nodes into central nodes and rest of the nodes non-central. As we are using DBpedia Spotlight to obtain information on the extracted entities, the ontology can be considered to share predicates with the ontology of DBpedia. The relationships and their counterparts in DBpedia’s syntax are depicted in the table in Fig. 3.

When populating the knowledge graph using the ontology, we are not setting any requirements or rules to the types of categories that might appear in the automated construction process. Every entity is considered as a type of organization, with the distinguishing feature being the type of industry the entity has. For instance, a government organization would have an industry indicating public service. The set of entity attributes for describing cyberattacks and events in our ontology were chosen as such to maintain readability and simplicity of the knowledge graph. It is also worth noting, that increasing the number of predicates for a given entity affects the network properties of the resulting knowledge graph. Naturally, the number of these predicates can be increased if the analyst requires other information within the boundaries of information available, but the current set serves as a backbone for the purpose of this study. The finalized result of the knowledge graph using the ontology presented here contains five types of nodes (entity, country, industry, product, attacker) and the five relationships described above. An example of a subset of the resulting knowledge graph is shown in Fig. 7.

### Measuring risk level

In addition to the situational awareness and human readability provided by the knowledge graph, we aim to quantify risk for the entities in the graph. Rather than trying to predict the occurrence of cyberattacks, our focus is on measuring risk from the relationship between sequential attack records. The level of risk in this study is measured from the network structure of the knowledge graph. As an attack is recorded in the form of an SVO-triple and a date, we can construct risk levels for the central nodes, which consist of industry and country nodes. Risk levels for the central nodes in the knowledge graphs are then used as a proxy for the connected entities via the linkages in the network structure.

In this initial model, we consider the risk $r_c(t)$ for a single central node *c* at time step *t* to be given by a sum of decaying exponential weights over the past events, such that

1 $$\begin{aligned} r_c(t) = \sum _{i}^{t} \exp {(i-t)}, \end{aligned}$$

where *t* denotes the time when the risk is calculated, and *i* is a time when an attack happened. The unit of time is considered as a parameter for adjusting the contributions of the past events to the current risk. After testing on our data for different values, we chose time to be in units of 30 days. The time step can be chosen for an appropriate duration, considering the type of data represented in the knowledge graph. For each attack-triple the time of reported occurrence is stored in the data structure. Should an attack be spread on multiple days, each day of the attack would be presented as a separate triple.

We also calculate the second central neighbors for the entity nodes by constructing a projection (see Fig. 6) of the network in such a way that the central nodes sharing entity nodes are connected in the projection. In addition to forming connections, the projection can be used to provide weights on the links between the central entities based on the number of shared entities. In the scope of this study we chose to consider every link with equal weight due to the fact that entities in the data are all part of the collected dataset and not an unbiased sample of entities in industries or countries. In an operational implementation of the framework the projection should be temporal and change over time in terms of the evolving amount of common entities between the central nodes. Constructing the projection allows us to investigate whether the risk propagates across the network and whether certain types of central nodes have more importance when considering the weights in the risk measures.

For a non-central entity *e* in the graph (i.e. an organization or a company), the risk level at a certain time step can be calculated from the neighboring central nodes by calculating a sum of the means

2 $$\begin{aligned} r_e = \overline{r_e(C)} + \overline{r_e(I)} + \overline{r_e(c)} + \overline{r_e(i)}, \end{aligned}$$

where $\overline{r_e(C)}$ denotes the mean of risk for the first neighbor country nodes, $\overline{r_e(I)}$ denotes the mean of risk for first neighbor industry nodes and *c* and *i* denote the risk for the second neighbor countries and industries in the projection, respectively. The second neighbors are considered to be the immediate neighbors of the central nodes *C* and *I* in the projection, *C* and *I* being connected to the focal entity *e* in the knowledge graph. We construct these risk measures into a dataset, in which for each day any non-central entity can be evaluated using a vector of these four values.

**Figure 4:**
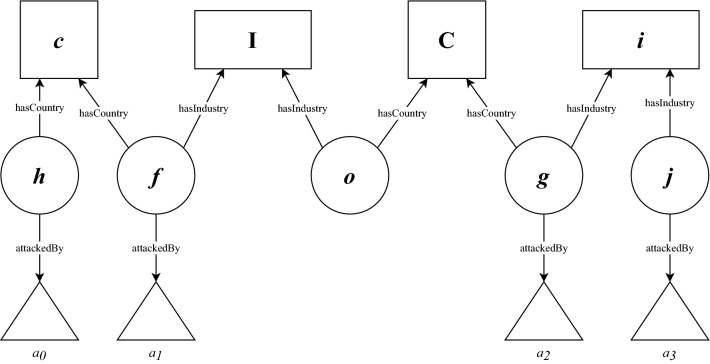
Example of the structure of the knowledge graph for calculating the heuristic risk value for the focal entity *o*. The focal entity is connected to industry *I* and country *C*. The rest of the graph is populated with entities *h*, *f*, *g* and *j* as well as industry *i* and country *c*. The recorded attacks to the entities are denoted from $a_{0}$ to $a_{3}$ in the order of occurrence at times $t_{0}$ to $t_{3}$.

In practice, the risk level shown in Eq. (2) is constructed as follows. Let us consider an organization *o* that is connected to a single industry node *I* and a single country node *C* in a random knowledge graph at time *t*. The structure of this example graph is shown in Fig. 4. To calculate the sum for $r_o$ we first consider the mean of the risk value depicted in Eq. (1) for the first neighbour nodes *I* and *C*. As the attacks to entities linked to the nodes *I* and *C* occur at times $t_1$ for attack $a_1$ and at $t_2$ for attack $a_2$, the risk values are $r_o(C) = \exp {(t_2 - t)}$ and $r_o(I) = \exp {(t_1 - t)}$, respectively. In a similar fashion, we calculate the risk for the second neighbour nodes, i.e. the nodes that share entities with the central nodes the focal entity is connected to, *i* and *c*. The risk measures for these nodes would be $r_o(c) = (\exp {(t_0-t)}+\exp {(t_1-t)})/2$ and $r_o(i) = (\exp {(t_2-t)}+\exp {(t_3-t)})/2$. Thus, the risk *r*_o_ would be $r_o = \exp {(t_2 - t)} + \exp {(t_1 - t)} + (\exp {(t_0-t)}+\exp {(t_1-t)})/2 + (\exp {(t_2-t)}+\exp {(t_3-t)})/2$. This risk for entity $o$ is not measuring the probability of an attack, but it is a measure depicting the recent incidents of attacks in the immediate and extended neighbourhood. We investigate the usefulness of this measure by using the components of the sum as a set of values.

### Experimental setup and analysis methods

We evaluate the knowledge mining framework by combining a dataset from Hackmageddon’s cyberattack timelines^38^ for each month from January 2017 to April 2021. For each article we crawled the original text whenever possible and processed its text through the NLP pipeline, extracting the attacked entity, the attacking entity and matching them with the entities recognized by DBpedia Spotlight. As the empirical data contains these fields already annotated by humans, we also compared the extracted entities to the fields in the original data and substituted missing text reports or entities from the annotated dataset to properly evaluate the graph analysis and risk module. Considering the shortcomings of classifying industries in a standard way or obtaining the operating countries for multi-national or lesser known organizations not present in DBpedia, we also add the annotated fields from the dataset in the knowledge graph as the industries and countries for the entities in addition to the ones obtained from DBpedia. In order to maintain the integrity of the dataset, we omit the rows where the victim is not specifically reported (i.e. various victims in multiple countries) or the countries or industries are not exact in a similar manner. The resulting nodes are resolved by comparing them to one another by using string similarity and joining sufficiently similar nodes. Processing the dataset using these methods resulted in a knowledge graph of 12,966 nodes and 18,476 edges. The filtered and processed data leaves us with 6825 SVO attack triples.

We construct the risk measure by first binning and ordering the recorded attacks and the triples into daily bins, after which we calculate the number of attacks towards the entities connected to the central nodes. The risk levels for each central node were calculated using the formula in Eq. (1). For each non-central entity illustrated in the resulting knowledge graph, we construct the averages of the first and second neighbor countries and industries in the graph into sets of four variables for each day, i.e. $\{\overline{r_e(C)}, \overline{r_e(I)}, \overline{r_e(c)}, \overline{r_e(i)}\}$. We split the dataset into “attack days” and “non-attack days” by letting the values for the attack days to be the values for the previous time step and sampling a non-attack day as a random day between the beginning of the dataset and the recorded attack date. By this process we obtain a dataset with 11,028 observations with equal amount of points in both classes.

To further interpret the usefulness of the constructed risk variables we perform a logistic regression and dimensionality reduction on the dataset of attack days and non-attack days. The objective of this analysis is to investigate the separability of the two classes and the relationship between the risk variables. These methods were implemented with the Scikit-learn library^46^. For the logistic regression classifier we split the constructed dataset into a training set and testing set with a 60–40 ratio at random. Fitting the logistic function to the training data allows us to evaluate the feasibility of the risk measures in estimating the likelihood of cyberattacks, even though the objective of this framework is not to predict exact attack dates. By fitting the classifier we also obtain the weights for the variables, which we can interpret in terms of their importance and mutual relationship. The last part of our evaluation was conducted using a principle component analysis (PCA) on the dataset to portray the attack days and non-attack days in two dimensions.

## Results and analysis on an experimental dataset

**Figure 5:**
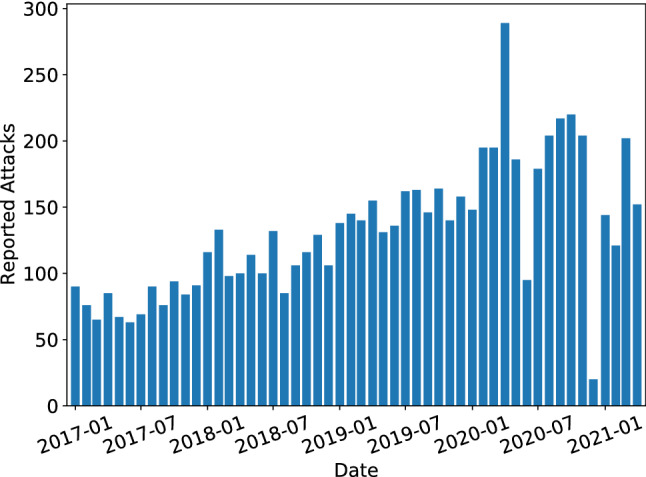
Number of monthly incidents in the filtered dataset. Records that did not produce a single coherent SVO-triple (subject–verb–object) for the attack were omitted to produce a consistent knowledge graph. It should be noted that the time of occurrence of an event (a triple) in the dataset can be wrongly recorded or reported long after the attack. Also, it is notable from the number of attacks that the reporting is not uniform and some months are much less populated than others, likely resulting from human error or bias.

**Figure 6:**
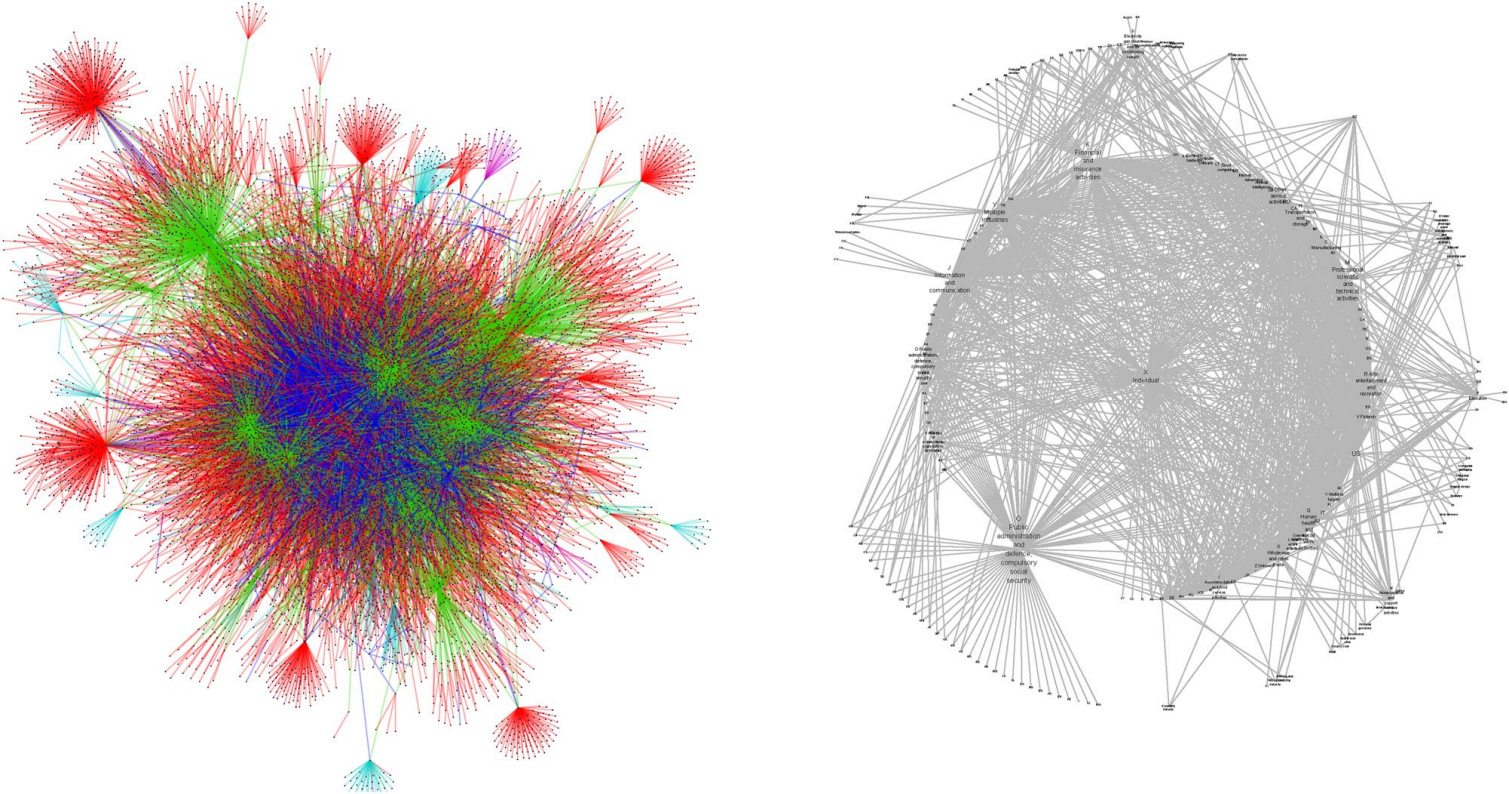
(Left) The resulting knowledge graph from the Hackmageddon dataset. The edges are coloured according to the interaction in the related triples such that red edges represent the attack triples (attackedBy), blue edges represent hasCountry, green edges represent hasIndustry, purple edges represent hasProduct triples, and turquoise edges represent hasParent triples. (Right) The projection of the central nodes used in the construction of the entity risk measures. The projection is constructed by linking central nodes sharing common neighbors such that the weight of every link is uniform regardless of the number of common neighbors.

**Figure 7:**
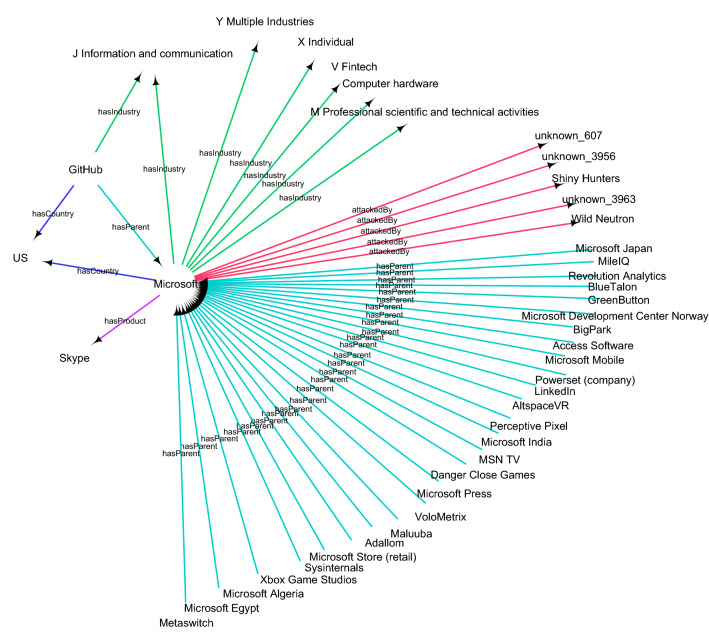
An example subset of the knowledge graph showing an egocentric network of the company Microsoft. The central entity is connected to the reported malicious entities (red links), the industries reported in the dataset as well as the ones obtained from DBpedia (green links), country (blue link), products (purple links) and child companies (turquoise links). The network is a subset of the knowledge graph with nodes and links of a single step from the focal node. The central nodes of this subset are connected to the focal node by green and blue links.

The frequency of reported attacks in the combined dataset is shown in Fig. 5. The number of reported attacks per month shows an increasing trend but the months of June and December in 2020 have significantly lower number of incidences, indicating that the reporting in the data can be incomplete. The nodes with the highest degree (i.e. the most connected to other nodes) are industries (public sector, healthcare) and countries (US, UK). The non-central nodes with the highest degree are the tech giants such as Google and Amazon and the users of their products such as the Android operating system. As we use the industry fields from the annotated dataset in addition to the ones obtained using DBpedia Spotlight, the network consists of a single connected component. The finalized knowledge graph based on the dataset from Hackmageddon cyberattack timelines from January 2017 to April 2021 and extraneous information from DBpedia is shown in Fig. 6 and a more descriptive subset of the same graph is shown in Fig. 7.

**Figure 8:**
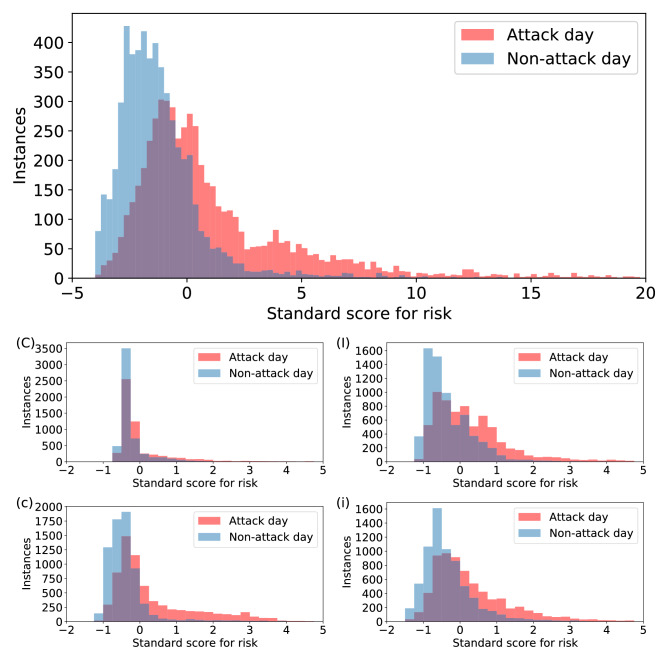
The distributions of resulting risk levels of attack and non-attack days in the knowledge graph. (Top) The sum of the four standardized variables, (Second row) Standard scores for risk in the first neighbor country node *C* and standard scores for average risk in first neighbor industry nodes *I*. (Third row) Standard scores for average risk in second neighbor countries *c* and second neighbor industries *i*. The two classes have equal number of observations.

The standardized distributions for the four variables in Eq. (2) are shown in Fig. 8. Overall, the distributions between the classes seem to differ from one another, the attack day distribution having a longer tail and more positive mean. It is notable that the distribution of the first neighbor’s (country) risk is very similar between the attack days, whereas the second neighbor’s risk shows a difference between the attack days and non-attack days. The differences between the two classes in the distributions of risk values show that there is some commonality within the classes.

**Figure 9:**
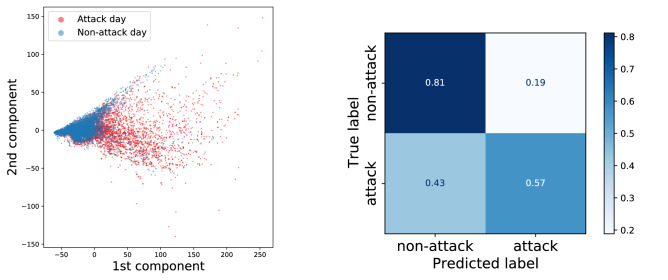
(Left) First two components of PCA dimensionality reduction on the risk data consisting of the four variables (*C*, *I*, *c* and *i*). The component loadings are shown in Table 1. Difference of the two classes in the plot indicates that a level of separability exists in the dataset. (Right) Confusion matrix obtained by training and testing a logistic regression classifier on the proposed risk measures. The overall accuracy of the classifier was around 0.69 and F1 score was 0.65.

Training a logistic regression classifier with standardized training and validation set constructed from the data results in around $69\%$ accuracy, which shows that there is, indeed, some relationship between the attacks in the network, at least in a temporal sense appearing as burstiness. The coefficients for the logistic regression (see Table 1) show that the first neighbour country has a very minor weight in the classifier function. The corresponding distribution *C* in Fig. 8 reinforces this as the two classes are highly overlapping. Interestingly, the coefficient of second neighbor country (*c*) is the highest, which could be interpreted as some countries being the catalysts or initial targets for chains of attacks. Also, the higher weight for *c* tells that multiple attacks to entities in a single country within a short time interval are not well represented in the analyzed dataset. The confusion matrix for the logistic regression is depicted in Fig. 9, showing the fractions of correctly and incorrectly predicted labels. As one would expect from the differences in the distributions of the constructed variables, the accuracy for correctly predicting non-attack days is higher (0.81) than correctly predicting the attack days (0.57).

**Table 1:** PCA component weights and coefficients from fitting a logistic regression to the data.

Variable	PCA 1st component	PCA 2nd component	Logistic regression coefficient
*C*	0.075	− 0.127	− 0.004
*I*	0.516	− 0.737	0.025
*c*	0.176	− 0.377	0.039
*i*	0.835	0.547	0.007

The variables are notated as first neighbor country (*C*), first neighbor industry (*I*), second neighbor country (*c*) and second neighbor industry (*i*).

Performing a dimensionality reduction with principle component analysis (see Fig. 9 left panel) results in components explaining 94% of the variance (83% and 11% for the two components). The component weights are shown in Table 1. These weights can be interpret as two different risk factors, industry-based and system-based risk. The industry-based risk in this situation can be reasoned from the higher factors for the industry nodes (*I* and *i*) in the first principal component and the system-based risk can be considered due to negative factors to all but second neighbour industry risk *i*. Lower factors for first neighbor country *C* in both components are apparent from the overlap in the variable’s distribution shown in Fig. 8.

## Discussion

As a whole, the advantages of the presented knowledge mining framework are its generality, explainability and extensibility. The generality allows us to consider different types of attacks and entities with limited information and resources. The information used to construct the strategic level graph with high abstraction is mostly available in online sources such as DBpedia and the extraction of the victim–attacker triple from unstructured data can be performed efficiently using the methods presented in this paper. The ontology is human-readable and easy to explain and can thus serve as a tool for communicating a large number of events and investigations with other people, such as decision and policy makers. As the present knowledge mining framework can be used to semi-automatically produce a contextual situational picture of the cyberspace and compute levels of risk for entities and industries, it could also serve as a tool for companies with limited resources on cyber intelligence and analysis. The rational for using this kind of framework to conducting investigations is its ability to conceptualize trends and similarities between attacks on a high abstraction level that does not require high level details or authorised knowledge about the entities or the entities’ systems. Thus, the metric of risk for an entity depicts the incidence of attacks to other entities with similar association or purpose. The framework can be extended to include information on software vulnerabilities, software used by the entities, and importance of entities in various supply-chain systems, moving the knowledge graph towards a more operational scope. However, the availability of such information is restricted and for the scope of this study we decided to keep the network structure human-readable by having only the essential nodes for describing general entities and incidents and relationships between them.

The analysis of the risk measure has shown that there can be some level of temporal and structural correlation between the recorded attacks. The distributions between the attack and non-attack days in the dataset differ from each other to a degree (see Fig. 8) and performing a logistic regression classification on the produced knowledge graph dataset yields a moderate accuracy (see Fig. 9). This reinforces the usefulness of our strategic level ontology, which assumes that similar entities have some common factors that are not always publicly reported and that similar companies are often targeted during some period of time. The relationship between different entities in the knowledge graph can be more complex than just surface level similarities and contain hidden variables, such as the used systems and protocols, which could explain some of the pathways between the various entities that are connected to different central nodes. Correlations between attacks and attacked entities in the knowledge graph can also be because the attackers focus on certain type of entities for their own reasons, or because recent vulnerabilities or data breaches have compromised entities with similar connections within the knowledge graph. The risk measures presented here are intended for evaluating our framework rather than investigating the real life risk. The results show that our framework has potential in formulating a measure of risk in addition to the capabilities on visualizing a large dataset for situational awareness and investigation.

There are also limitations to this framework. The risk measure used in this study is based on the network structure of the resulting knowledge graph and thus the design choices could have impact on the variables constructed. The results can be biased and affected by numerous sources, such as human error and bias in the reporting of the incidents and collecting the incidents to the dataset. The constructed knowledge graph is hardly a ground truth of all cyberattacks due to lack of reporting or detection. The language of the collected dataset can impose a limitation on geographical areas, where the reporting language is different from the one used in the implementation of the framework. Other biases can rise from the accuracy of processing the unstructured data into a knowledge graph as well as the types of extra information added to the graph, such as the types of industry nodes. The generality of the industries have a direct effect on the structure of the graph and related properties. Also, the accuracy for recording and reporting a cyber-related incident can vary in public online sources. The discovery of an attack can be late and thus the accurate time might not be reported correctly. This is a direct limitation to the estimation of risk in the framework. For instance, the dates reported in the empirical dataset used in this study vary in accuracy with some dates reported days later than the incident in question. It should also be noted that online reports from unauthorized sources and written by journalists of various backgrounds can contain misinformation or disinformation. Unreliability of the data is a limitation of this framework, potentially affecting the measurement of the risk. Better credibility and quality of the data obtained can be achieved with additional manual labor, such as verification. Using a set of trusted sources, such as national or international cyber security authorities, can also mitigate inaccuracies and false reports. The relevant information extracted from the reports is the SVO triple, which is not affected by possibly inaccurate reporting of attack types, for instance. The required relevant information is the correct victim entity and a date.

Within the information extraction pipeline there are two primary sources of possible inaccuracies. The first involves information from the dependency parser. This was done by using spaCy’s trained pipelines and is, therefore, indirectly depended on the performance of the in-built neural network predictions. The second source of inaccuracy is the scoring method used for ranking of triples. While we achieve a moderate accuracy of $60\%$ (and $83\%$ for target to lie within the top three ranks) by this method, the results indicate the importance of inclusion of the target score. Just using the order of appearance or frequency lowers the accuracy by $10\%$. Also, as noted earlier the baseline accuracy would be much smaller.

For evaluating the risk measure, we used the annotated dataset when the information was not available. There were a number of such situations, either because of preventive measures of the websites or the URL being deprecated after a period of time. Without using the annotated fields, the number of entities and observations would be lower and the graph structure would be different without the industry categories. Also, the number of industries and entities would be different in a real world application of our knowledge graph based framework, as all of the entities in the knowledge graph of this study were either attacked at some point or related to these entities as parent or child companies. A larger number of entities would have an effect on the risk measures for second neighbour countries and industries by affecting the projection. The classifier used to evaluate the risk metric was trained and evaluated on an evenly balanced dataset, thus the accuracy should not be considered to predict the attack date from any possible set of dates, but as a demonstration that binary classification using the risk variables yields better accuracy than random guessing.

## Conclusions

In this study we have presented a novel knowledge graph based framework for constructing a strategic level mapping of the current and past cyberattacks from unstructured reports in the open online sources and demonstrated the capabilities of the resulting knowledge graph in terms of communicating events and constructing measures for risk. The aim of this framework is to structure textual data into computer-readable and computable form, facilitate measures for risk and help expert analysts to process and view a large amount of reports in an automated manner. The pipeline combines methods and techniques from NLP and complex networks, starting with scraping and retrieving of articles from online sources, extracting relevant entities and the correct subject–verb–object or SVO-triples on the attacked entities and the attacking actors, and finalizing by constructing a knowledge graph with an ontology consisting of five types of nodes and relationships (see Fig. 3). We have implemented the pipeline and the related algorithms in Python 3.7 programming language and created a knowledge graph using the pre-annotated dataset from Hackmageddon that contains over 7000 recorded attacks between January 2017 and April 2021 (see Fig. 6). With this knowledge graph we have also constructed a measure of risk, which is based on a decaying time-based function and the network structure of the knowledge graph.

We believe that the methods and results of this study can help cyber-analysts to perform their investigations more efficiently in the future as the amount of new information is increasing faster than the number of experts available at any time. In our future research, we plan to improve the methods for information extraction from unstructured sources for better accuracy and generalization, which would improve the reliability and validity of the knowledge graph as well as provide a possibility for better automation in terms of facilitating the framework as a continuous process. Constructing language-agnostic tools for this task would also solve the problem of having a limited focus on certain parts of the world. As discussed previously, adding new information from other sources, such as system information of entities and various vulnerability databases, could increase the accuracy of the risk model, should such information be available. This would also allow to conduct simulations and “what–if” type scenarios on the knowledge graph, possibly being able to show more microscopic trends or campaigns. In addition to high-level scenarios, such as common infrastructure, one could utilize the vast work done in the field of models and simulations for vulnerability analysis^47,48^. Also, joining the system level technical information and accurate industry information to the framework could allow categorization of the events into different aspects of the society such as political, economical and military-operations.

## Data Availability

The datasets analysed during the current study are available in the Hackmageddon website^38^. The combined dataset and code used in this study are available from the corresponding author on reasonable request.

## References

[1] Forum, W. E. The global risks report 2021. https://www.weforum.org/reports/the-global-risks-report-2021. Online; Accessed 10 January 2021 (2021).

[2] CERT-EU. Latest news. https://cert.europa.eu/cert/filteredition/en/CERT-LatestNews.html. Online; Accessed 13 January 2021 (2021).

[3] Liu K (2022). Recent progress of using knowledge graph for cybersecurity. Electronics.

[4] Li, R., Dai, W., He, S., Chen, X. & Yang, G. A knowledge graph framework for software-defined industrial cyber-physical systems. In *IECON 2019-45th Annual Conference of the IEEE Industrial Electronics Society*, vol. 1, 2877–2882 (IEEE, 2019).

[5] Piplai A (2020). Creating cybersecurity knowledge graphs from malware after action reports. IEEE Access.

[6] Li, K., Zhou, H., Tu, Z. & Feng, B. Cskb: A cyber security knowledge base based on knowledge graph. In *International Conference on Security and Privacy in Digital Economy*, 100–113 (Springer, 2020).

[7] Böhm F, Menges F, Pernul G (2018). Graph-based visual analytics for cyber threat intelligence. Cybersecurity.

[8] Barnum S (2012). Standardizing cyber threat intelligence information with the structured threat information expression (stix). Mitre Corp..

[9] Syed, Z., Padia, A., Finin, T., Mathews, L. & Joshi, A. Uco: A unified cybersecurity ontology. In *UMBC Student Collection* (2016).

[10] Iannacone, M. *et al.* Developing an ontology for cyber security knowledge graphs. In *Proceedings of the 10th Annual Cyber and Information Security Research Conference*, 1–4 (2015).

[11] Joshi, A., Lal, R., Finin, T. & Joshi, A. Extracting cybersecurity related linked data from text. In *2013 IEEE Seventh International Conference on Semantic Computing*, 252–259 (IEEE, 2013).

[12] Auer, S. *et al.* Dbpedia: A nucleus for a web of open data. In *Proceedings of the 6th International The Semantic Web and 2nd Asian Conference on Asian Semantic Web Conference, ISWC’07/ASWC’07*, 722–735 (Springer, Berlin, Heidelberg, 2007).

[13] Ehrlinger, L. & Wöß, W. Towards a definition of knowledge graphs. In *SEMANTiCS (Posters, Demos, SuCCESS)*, vol. 48, 2 (2016).

[14] Duan, Y. *et al.* Specifying architecture of knowledge graph with data graph, information graph, knowledge graph and wisdom graph. In *2017 IEEE 15th International Conference on Software Engineering Research, Management and Applications (SERA)*, 327–332 (IEEE, 2017).

[15] Finkel, J. R., Grenager, T. & Manning, C. D. Incorporating non-local information into information extraction systems by Gibbs sampling. In *Proceedings of the 43rd Annual Meeting of the Association for Computational Linguistics (ACL’05)*, 363–370 (2005).

[16] Shen, Y., Colloc, J., Jacquet-Andrieu, A., Guo, Z. & Liu, Y. Constructing ontology-based cancer treatment decision support system with case-based reasoning. In *International Conference on Smart Computing and Communication*, 278–288 (Springer, 2017).

[17] Rotmensch M, Halpern Y, Tlimat A, Horng S, Sontag D (2017). Learning a health knowledge graph from electronic medical records. Sci. Rep..

[18] Auer, S. *et al.* Towards a knowledge graph for science. In *Proceedings of the 8th International Conference on Web Intelligence, Mining and Semantics*, 1–6 (2018).

[19] Georgescu TM, Smeureanu I (2017). Using ontologies in cybersecurity field. Inf. Econom..

[20] National Institute of Standards and Technology. National vulnerability database (NVD).

[21] MITRE Corporation. Common vulnerabilities and exposures (CVE).

[22] MITRE Corporation. Common weakness enumeration (CWE).

[23] Mavroeidis, V. & Bromander, S. Cyber threat intelligence model: An evaluation of taxonomies, sharing standards, and ontologies within cyber threat intelligence. In *2017 European Intelligence and Security Informatics Conference (EISIC)*, 91–98 (IEEE, 2017).

[24] Rastogi, N., Dutta, S., Zaki, M. J., Gittens, A. & Aggarwal, C. Malont: An ontology for malware threat intelligence. In *International Workshop on Deployable Machine Learning for Security Defense*, 28–44 (Springer, 2020).

[25] Komárková, J., Husák, M., Laštovička, M. & Tovarňák, D. Crusoe: Data model for cyber situational awareness. In *Proceedings of the 13th International Conference on Availability, Reliability and Security*, 1–10 (2018).

[26] Heinbockel, W., Noel, S. & Curbo, J. Mission dependency modeling for cyber situational awareness. In *NATO IST-148 Symposium on Cyber Defence Situation Awareness*, 1–14 (2016).

[27] Noel, S., Harley, E., Tam, K. H., Limiero, M. & Share, M. Cygraph: Graph-based analytics and visualization for cybersecurity. In *Handbook of Statistics*, vol. 35, 117–167 (Elsevier, 2016).

[28] Schäfer, M. *et al.* Blackwidow: Monitoring the dark web for cyber security information. In *2019 11th International Conference on Cyber Conflict (CyCon)*, vol. 900, 1–21 (IEEE, 2019).

[29] Tavabi, N., Goyal, P., Almukaynizi, M., Shakarian, P. & Lerman, K. Darkembed: Exploit prediction with neural language models. In *Proceedings of the AAAI Conference on Artificial Intelligence*, vol. 32 (2018).

[30] Mittal, S., Das, P. K., Mulwad, V., Joshi, A. & Finin, T. Cybertwitter: Using twitter to generate alerts for cybersecurity threats and vulnerabilities. In *2016 IEEE/ACM International Conference on Advances in Social Networks Analysis and Mining (ASONAM)*, 860–867 (IEEE, 2016).

[31] Mittal, S., Joshi, A. & Finin, T. Cyber-all-intel: An AI for security related threat intelligence. arXiv preprint arXiv:1905.02895 (2019).

[32] Neil, L., Mittal, S. & Joshi, A. Mining threat intelligence about open-source projects and libraries from code repository issues and bug reports. In *2018 IEEE International Conference on Intelligence and Security Informatics (ISI)*, 7–12 (IEEE, 2018).

[33] Jia Y, Qi Y, Shang H, Jiang R, Li A (2018). A practical approach to constructing a knowledge graph for cybersecurity. Engineering.

[34] Kejriwal, M. & Szekely, P. Information extraction in illicit web domains. In *Proceedings of the 26th international conference on world wide web*, 997–1006 (2017).

[35] Honnibal, M., Montani, I., Van Landeghem, S. & Boyd, A. *spaCy: Industrial-Strength Natural Language Processing in Python*. 10.5281/zenodo.1212303 (2020).

[36] Daiber, J., Jakob, M., Hokamp, C. & Mendes, P. N. Improving efficiency and accuracy in multilingual entity extraction. In *Proceedings of the 9th International Conference on Semantic Systems (I-Semantics)* (2013).

[37] Hagberg, A., Swart, P. & S Chult, D. Exploring network structure, dynamics, and function using networkx. Tech. Rep., Los Alamos National Lab.(LANL), Los Alamos, NM (United States) (2008).

[38] Passeri, P. Hackmageddon. https://www.hackmageddon.com/ (2021). Accessed 14 August 2021.

[39] Rosenfeld, B. & Feldman, R. Ures: an unsupervised web relation extraction system. In *Proceedings of the COLING/ACL 2006 Main Conference Poster Sessions*, 667–674 (2006).

[40] Pingle, A. *et al.* Relext: Relation extraction using deep learning approaches for cybersecurity knowledge graph improvement. In *Proceedings of the 2019 IEEE/ACM International Conference on Advances in Social Networks Analysis and Mining*, 879–886 (2019).

[41] Stewart, M., Enkhsaikhan, M. & Liu, W. Icdm 2019 knowledge graph contest: Team uwa. In *2019 IEEE International Conference on Data Mining (ICDM)*, 1546–1551, 10.1109/ICDM.2019.00205 (2019).

[42] D’Souza, S. Parser extraction of triples in unstructured text. arXiv preprint arXiv:1811.05768 (2018).

[43] Etzioni, O., Fader, A., Christensen, J., Soderland, S. & Mausam, M. Open information extraction: The second generation. In *IJCAI*, vol. 11, 3–10 (2011).

[44] Hearst, M. A. Automatic acquisition of hyponyms from large text corpora. In *Coling 1992 volume 2: The 15th International Conference on Computational Linguistics* (1992).

[45] Wolf, T. State-of-the-art neural coreference resolution for chatbots. http://medium.com/huggingface/state-of-the-art-neural-coreference-resolution-for-chatbots-3302365dcf30 (2017). Accessed 07 July 2021.

[46] Pedregosa F (2011). Scikit-learn: Machine learning in Python. J. Mach. Learn. Res..

[47] Ficco M, Choraś M, Kozik R (2017). Simulation platform for cyber-security and vulnerability analysis of critical infrastructures. J. Comput. Sci..

[48] Kavak H (2021). Simulation for cybersecurity: state of the art and future directions. J. Cybersecur..

